# Studies on the cold tolerance of ratoon ‘Chaling’ common wild rice

**DOI:** 10.1186/s40659-020-00276-5

**Published:** 2020-02-18

**Authors:** Mengliang Xu, Xiangzhen Li, Xiang Mo, Siyu Tu, Yanchun Cui, Daichang Yang

**Affiliations:** 1 Hunan Provincial Key Laboratory of Crop Sterility Mechanism and Sterile Germplasm Resources Innovation, State Key Laboratory of Developmental Biology of Freshwater Fish, College of Life Sciences, Hunan Normal University, Changsha, 410081 China; 2 Key Laboratory of Agro-ecological Processes in Subtropical Region, Institute of Subtropical Agriculture, Chinese Academy of Sciences, Changsha, 410125 China; 3 State Key Laboratory of Hybrid Rice, College of Life Sciences, Wuhan University, Wuhan, 430072 China

**Keywords:** *Oryza rufipogon* Griff., *Oryza sativa* L., Ratoon rice, Cold stress, Overwinter, Survival rate

## Abstract

**Background:**

Rice is the staple food of many people around the world. However, most rice varieties, especially widely grown *indica* varieties and hybrids, are sensitive to cold stress. In order to provide a basis for the utilization of a common wild rice (CWR, *Oryza rufipogon* Griff.) named ‘Chaling’ CWR in cold-tolerant rice breeding and deepen the understanding of rice cold tolerance, the cold tolerance of ratoon ‘Chaling’ CWR was studied under the stress of the natural low temperature in winter in Changsha, Hunan province, China, especially under the stress of abnormal natural low temperature in Changsha in 2008, taking other ratoon CWR accessions and ratoon cultivated rice phenotypes as control.

**Results:**

The results showed that ratoon ‘Chaling’ CWR can safely overwinter under the natural conditions in Changsha (28° 22′ N), Hunan province, China, which is a further and colder northern place than its habitat, even if it suffers a long-term low temperature stress with ice and snow. In 2008, an extremely cold winter appeared in Changsha, i.e., the average daily mean temperature of 22 consecutive days from January 13 to February 3 was − 1.0 °C, and the extreme low temperature was − 4.7 °C. After subjected to this long-term cold stress, the overwinter survival rate of ratoon ‘Chaling’ CWR was 100%, equals to that of ratoon ‘Dongxiang’ CWR which is northernmost distribution in the word among wild rice populations, higher than those of ratoon ‘Fusui’ CWR, ratoon ‘Jiangyong’ CWR, and ratoon ‘Liujiang’ CWR (63.55–83.5%) as well as those of ratoon ‘Hainan’ CWR, ratoon ‘Hepu’ CWR, and all the ratoon cultivated rice phenotypes including 3 *japonica* ones, 3 *javanica* ones, and 5 *indica* ones (0.0%).

**Conclusions:**

The results indicate that ratoon ‘Chaling’ CWR possesses strong cold tolerance and certain freezing tolerance.

## Background

**Fig. 1:**
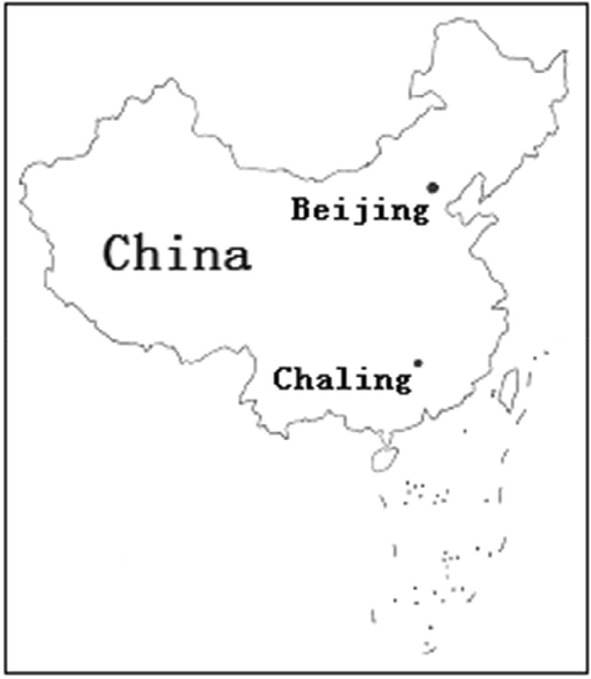
The location (26° 50′ N) of the habitat of Chaling CWR (*Oryza rufipogon* Griff.), Chaling county, Hunan province, China

Rice is the staple food of many people all over the world. However, most of its varieties, especially those of widely grown *indica* varieties and *indica* hybrids, are sensitive to cold stress. Cold stress can seriously influence the rice production seasonally and regionally, and is also a main factor that limits the distribution of rice. So enhancing the cold tolerance of rice cultivars, especially *indica* cultivars is desired in order to alleviate the impact of cold stress on them. Developing rice cold tolerance varieties by using the cold tolerant wild rice accessions via conventional breeding or molecular breeding is an effective measure to enhance the cold tolerance of rice cultivars. CWR is the close relative species of common cultivated rice (CCR, *Oryza sativa* L.) or Asian cultivated rice. Although many agronomic traits of CWR are inferior to those of CCR, the genetic polymorphism of CWR is richer than that of CCR [1–4]. Among CWR accessions there are some superior resources which possess excellent or specific agronomic traits like resistance to pest and disease, tolerance to abiotic stress, high yield quantitative trait locus/loci (QTL), etc. [5–8]. Therefore, CWR is an important genetic rice germplasm, and possess important value of protection, research, and utilization. ‘Chaling’ CWR is one of the CWR populations. It is distributed in a wetland named “Huli” (26° 50′ N), Yaoshui town, Chaling county, Hunan province, China (Fig. 1) [9, 10]. More importantly, it is one of two wild rice populations grown northernmost in the word (the other population is ‘Dongxiang’ CWR, distributed in Dongxiang county, Jiangxi province, China, 28° 14′ N). Some achievements have been made on the studies of its geographical distribution and ecological environment characteristics, living habits, botanical characteristics and systematic classification, resistance to disease and pest, tolerance to abiotic stress, and photosynthetic characteristics [9–15]. However, the study on its cold tolerance is not enough in that only the cold tolerance of its seedling was evaluated [13], while the similar study of the other CWR populations, especially ‘Dongxiang’ CWR, is relatively more [16–19]. This study aims to evaluate the cold tolerance of ratoon ‘Chaling’ CWR by investigating its survival rate after overwintering in Changsha, so as to provide a basis for its utilization, and deepen the understanding of rice cold tolerance.

## Results

**Fig. 2:**
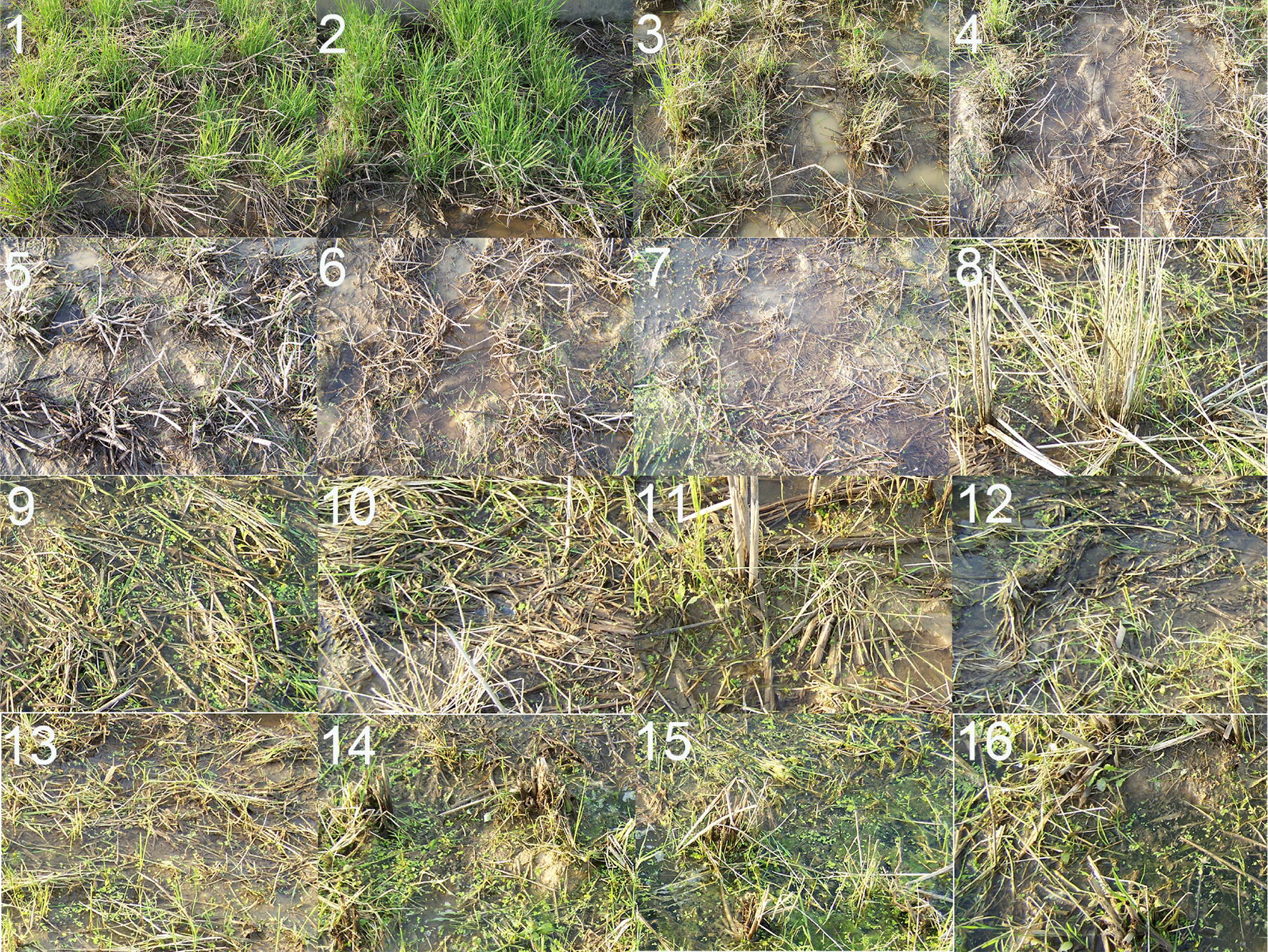
Phenotypic comparison between ratoon ‘Chaling’ CWR and other ratoon CWR accessions or different ratoon CCR varieties after overwinter in natural condition in Changsha, Hunan province, China (2008). 1 ratoon ‘Chaling’ CWR, 2 ratoon ‘Dongxiang’ CWR, 3 ratoon ‘Fusui’ CWR, 4 ratoon ‘Jiangyong’ CWR, 5 ratoon ‘Liujiang’ CWR, 6 ratoon ‘Hepu’ CWR, 7 ratoon ‘Hainan’ CWR, 8–10 ratoon *japonica* cultivars (Nipponbare, Qiuguang, and Dongnong 416), 11–13 ratoon *javanica* cultivars (01 Zhao 001, 01 Zhao 085, and 01 Zhao 092), 14–16 ratoon *indica* cultivars (Nanjing11, 93–11, and Liangyoupeijiu). Note: The average daily mean temperature of 22 consecutive days from January 13, 2008 to February 3, 2008 was − 1.0 °C, and the extreme low temperature was − 4.7 °C. Taken on April 24, 2008

“Chaling” CWR has the ability of overwintering in its native habitat (26° 50′ N), showing perennial living habits. It regenerates seedlings from the axillary buds of overwintering stem node in early March, heads in mid-September, and get mature in mid-October every year [10]. After we introduced it to Changsha (28° 22′ N) from its native habitat in 2002, although it has moved northward beyond the northernmost limit of the natural distribution of wild rice in the world (Dongxiang county, Jiangxi province, China, 28° 14′ N), it still shows the ability to overwinter, and it has been successfully overwintering in Changsha under natural conditions for 16 consecutive years. Especially in 2008, there was a rare long-term low-temperature climate with snow and ice in Changsha, Hunan province, China. The temperature in Changsha in mid-January, late January, and early February was abnormally low (Table 1). During the 22 consecutive days from January 13 to February 3 in 2008 in Changsha, the average daily mean temperature is − 1.0 °C, the average daily minimum temperature is − 1.7 °C, the average daily maximum temperature is 2.3 °C, and the extreme low temperature is − 4.7 °C. After this severe cold, all the ratoon ‘Chaling’ CWR plants survived as ratoon ‘Dongxiang’ CWR plants in our experimental paddy field, however some ratoon ‘Fusui’ CWR plants, ratoon ‘Jiangyong’ CWR plants, and ratoon ‘Liujiang’ CWR plants died while all the ratoon ‘Hainan’ CWR plants, ratoon ‘Hepu’ CWR plants, and all the cultivated rice plants (including rice plants of 3 *japonica* varieties, 3 *javanica* varieties, and 5 *indica* varieties) died (Table 2). After overwintering, with the rise in temperature, the regenerated seedlings on the stems of all the survival ratoon CWR plants grew well, and many new well-grown regenerated seedlings could be produced. However, the number of new well-grown regenerated seedlings on the stems of ratoon ‘Chaling’ CWR plants and ratoon ‘Dongxiang’ CWR plants was much more than that of the other survival ratoon CWR plants, i.e., they could regenerate 40–50 seedlings per plants while the other survival ratoon CWR plants could regenerate 1–30 seedlings per plants (Fig. 2). The results indicate that ratoon ‘Chaling’ CWR has strong cold tolerance and certain freezing tolerance.

**Table 1:** The air temperature from January to March at Changsha, Hunan province, China in 2008, 2010, and 2011 (°C)

Year	Month	A period of ten days	^①^ADT_mean_ (°C)	^②^ADT_min._ (°C)	^③^ADT_max._ (°C)	^④^T_min._ (°C)	^⑤^T_max._ (°C)
2008	Jan.	Early	8.38	4.09	14.43	− 0.9	19.5
		Middle	0.29	− 0.28	1.42	− 1.8	7.8
		Late	− 1.43	− 1.96	− 0.52	− 3.2	1.5
	Feb.	Early	1.46	− 1.20	4.89	− 4.7	7.3
		Middle	6.18	2.82	10.30	− 0.9	15.0
		Late	9.17	5.56	14.64	1.4	23.0
	March	Early	13.01	8.89	19.07	7.3	23.7
		Middle	16.75	13.90	21.55	11.9	27.0
		Late	15.71	12.27	20.12	9.4	27.2
2010	Jan.	Early	5.06	3.00	8.49	− 1.7	16.2
		Middle	7.97	4.38	13.01	− 0.1	22.1
		Late	6.79	5.38	8.97	1.6	12.7
	Feb.	Early	7.21	6.13	9.03	3.4	15.5
		Middle	3.80	1.44	7.02	− 1.3	17.8
		Late	16.64	12.20	23.21	6.9	30.6
	March	Early	5.12	3.31	8.29	− 1.1	21.2
		Middle	16.74	12.11	22.5	3.9	28.6
		Late	15.23	11.56	19.88	7.0	26.4
2011	Jan.	Early	1.28	− 0.17	3.29	− 2.2	5.2
		Middle	2.41	0.57	5.14	− 2.7	9.5
		Late	2.41	0.58	4.47	− 1.4	7.7
	Feb.	Early	9.92	5.41	16.66	0.2	24.3
		Middle	5.84	3.55	9.16	0.9	14.8
		Late	10.61	8.13	14.68	3.0	22.3
	March	Early	8.35	5.65	12.21	0.8	19.4
		Middle	11.73	8.85	16.19	4.1	20.1
		Late	12.48	8.97	16.78	5.9	24.4

1) ① average daily mean temperature; ② average daily minimum temperature; ③ average daily maximum temperature; ④ minimum temperature; ⑤ maximum temperature. 2) Based on data provided by China Meteorological Information Center

**Table 2:** Comparison of survival rates between ratoon ‘Chaling’ CWR and other ratoon CWR accessions or different ratoon CCR phenotypes after overwinter in natural condition in Changsha, Hunan province, China (2008)

Phenotypes or accessions	Classification	Number of plants before winter	Number of plants after winter	Overwinter survival rates (%)
‘Chaling’ CWR	*O. rufipongon*	20.0 ± 0.0 a	20.0 ± 0.0 a	100.0 ± 0.0 a
‘Dongxiang’ CWR	*O. rufipongon*	20.0 ± 0.0 a	20.0 ± 0.0 a	100.0 ± 0.0 a
‘Fusui’ CWR	*O. rufipongon*	20.0 ± 0.0 a	16.7 ± 2.1 b	83.3 ± 10.4 b
‘Jiangyong’ CWR	*O. rufipongon*	20.0 ± 0.0 a	14.3 ± 1.5 bc	71.7 ± 7.6 bc
‘Liujiang’ CWR	*O. rufipongon*	20.0 ± 0.0 a	12.7 ± 1.2 c	63.3 ± 5.8 c
‘Hepu’ CWR	*O. rufipongon*	20.0 ± 0.0 a	0.0 ± 0.0 d	0.0 ± 0.0 d
‘Hainan’ CWR	*O. rufipongon*	20.0 ± 0.0 a	0.0 ± 0.0 d	0.0 ± 0.0 d
Nipponbare	*O. sativa* ssp. *japonica*	20.0 ± 0.0 a	0.0 ± 0.0 d	0.0 ± 0.0 d
Qiuguang	*O. sativa* ssp. *japonica*	20.0 ± 0.0 a	0.0 ± 0.0 d	0.0 ± 0.0 d
Dongnong 416	*O. sativa* ssp. *japonica*	20.0 ± 0.0 a	0.0 ± 0.0 d	0.0 ± 0.0 d
01 Zhao 001	*O. sativa* ssp. *javanica*	20.0 ± 0.0 a	0.0 ± 0.0 d	0.0 ± 0.0 d
01 Zhao 085	*O. sativa* ssp. *javanica*	20.0 ± 0.0 a	0.0 ± 0.0 d	0.0 ± 0.0 d
01 Zhao 092	*O. sativa* ssp. *javanica*	20.0 ± 0.0 a	0.0 ± 0.0 d	0.0 ± 0.0 d
Nanjing11	*O. sativa* ssp. *indica*	20.0 ± 0.0 a	0.0 ± 0.0 d	0.0 ± 0.0 d
Guangluai 4	*O. sativa* ssp. *indica*	20.0 ± 0.0 a	0.0 ± 0.0 d	0.0 ± 0.0 d
93-11	*O. sativa* ssp. *indica*	20.0 ± 0.0 a	0.0 ± 0.0 d	0.0 ± 0.0 d
Liangyoupeijiu	*O. sativa* ssp. *indica*	20.0 ± 0.0 a	0.0 ± 0.0 d	0.0 ± 0.0 d
Peiai 64S	*O. sativa* ssp. *indica*	20.0 ± 0.0 a	0.0 ± 0.0 d	0.0 ± 0.0 d

The data in the table are mean ± standard deviation (SD). The means with the same letters within the same column denote no significant difference (P ≥ 0.05) among genotypes or accessions, while the means with the different letters denote significant difference (Duncan test, P < 0.05)

In 2010 and 2011, the cold tolerance of ratoon ‘Chaling’ CWR was also studied by pot experiment in Changsha. Compared with the temperature in middle and late January, and early February of 2008 in Changsha, the temperature in the same period of 2010 was higher (Table 1), however it was still cold stress to CWR and CCR. Under this cold stress, not only all the ratoon ‘Chaling’ CWR plants and ratoon ‘Dongxiang’ CWR plants survived, all the ratoon ‘Liujiang’ CWR plants and ‘Fusui’ CWR plants survived too, while ratoon ‘Jiangyong’ CWR plants still partially survived, and all the other ratoon plants including ‘Hainan’ CWR plants, ‘Hepu’ CWR plants, and all the CCR plants still died (Table 3). In the same period of 2011, although there was no the same cold as in 2008, the temperature was also very low for the average daily mean temperature was around 2 °C in the early, middle and late January, which was close to the temperature in the middle and late January, and early February of 2008 (Table 1). The test result of survival rates of ratoon CWR and CCR plants is identical with that in 2008, i.e., all the ratoon ‘Chaling’ CWR plants and ratoon ‘Dongxiang’ CWR plants successfully survived over the winter in Changsha, and a part of ratoon ‘Liujiang’ CWR plants, ‘Fusui’ CWR plants, and ‘Jiangyong’ CWR plants successfully survived, but all the ratoon ‘Hainan’ CWR plants, ‘Hepu’ CWR plants, and all the test ratoon CCR plants couldn’t survive, and eventually died (Table 3). The results of the pot experiment in these 2 years either indicated that the cold tolerance of ratoon ‘Chaling’ CWR is very strong.

**Table 3:** Comparison of survival rates between ratoon ‘Chaling’ CWR and other ratoon CWR accessions or different ratoon CCR phenotypes after overwinter in natural condition in Changsha, Hunan province, China (2010, 2011)

Phenotypes or accessions	Classification	The year of 2010			The year of 2011		
		Number of plants before Winter	Number of plants after Winter	Overwinter survival rates (%)	Number of plants before Winter	Number of plants after Winter	Overwinter survival rates (%)
‘Chaling’ CWR	*O. rufipongon*	10	10	100.0	10	10	100.0
‘Dongxiang’ CWR	*O. rufipongon*	10	10	100.0	10	10	100.0
‘Liujiang’ CWR	*O. rufipongon*	10	10	100.0	10	8	80.0
‘Fusui’ CWR	*O. rufipongon*	10	10	100.0	10	6	60.0
‘Jiangyong’ CWR	*O. rufipongon*	10	7	70.0	10	6	60.0
‘Hepu’ CWR	*O. rufipongon*	10	0	0.0	10	0	0.0
‘Hainan’ CWR	*O. rufipongon*	10	0	0.0	10	0	0.0
Nipponbare	*O. sativa* ssp. *japonica*	10	0	0.0	10	0	0.0
Qiuguang	*O. sativa* ssp. *japonica*	10	0	0.0	10	0	0.0
Dongnong 416	*O. sativa* ssp. *japonica*	10	0	0.0	10	0	0.0
01 Zhao 001	*O. sativa* ssp. *javanica*	10	0	0.0	10	0	0.0
01 Zhao 085	*O. sativa* ssp. *javanica*	10	0	0.0	10	0	0.0
01 Zhao 092	*O. sativa* ssp. *javanica*	10	0	0.0	10	0	0.0
Nanjing11	*O. sativa* ssp. *indica*	10	0	0.0	10	0	0.0
Guangluai 4	*O. sativa* ssp. *indica*	10	0	0.0	10	0	0.0
93-11	*O. sativa* ssp. *indica*	10	0	0.0	10	0	0.0
Liangyoupeijiu	*O. sativa* ssp. *indica*	10	0	0.0	10	0	0.0
Peiai 64S	*O. sativa* ssp. *indica*	10	0	0.0	10	0	0.0

## Discussion

In this study, the cold tolerance of ratoon ‘Chaling’ CWR was studied compared with the other ratoon CWR accessions and ratoon CCR varieties under the natural low temperature in winter in Changsha, Hunan province, China, especially under the long-term low temperature stress in 2008. The results showed that ratoon ‘Chaling’ CWR can safely overwinter in Changsha, just like ratoon ‘Dongxiang’ CWR, and its overwintering ability is obviously stronger than those of other ratoon CWR accessions with low distribution latitude and all ratoon CCR test varieties including typical *japonica* and *indica* rice varieties and several *javanica* rice varieties. As for question of how strong of its cold-tolerant ability, this study didn’t give a clear answer. It can be determined by the test in the higher latitude area or high altitude area combined with the sub-zero temperature control experiment. However, through this study, it is clear that under field conditions, even if ratoon ‘Chaling’ CWR was subjected to long-term low-temperature with ice and snow (the average daily mean temperature for continuous 22 days is − 1.0 °C, the average daily minimum temperature is − 1.7 °C, the average daily maximum temperature is 2.3 °C, and the extreme low temperature is − 4.7 °C), it can still safely overwinter, which is enough to support the conclusion that ratoon ‘Chaling’ CWR possesses strong cold tolerance and certain freeze resistance. Additionally, we found that the photosynthetic characteristics of ‘Chaling’ CWR under cold stress (15 °C) are superior to those of the standard rice cultivars including *indica* ‘Guangluai 4’, *indica* ‘Y-Liangyou 1’ (a Chinese hybrid rice with super high grain yield), and *japonica* ‘Nipponbare’ at their tiller stages [11], and ‘Chaling’ CWR grows better than the standard rice cultivars under the cold stress. Therefore, the authors believe that ‘Chaling’ CWR is a valuable rice cold-tolerant germplasm which has potential utilization value in improving the cold tolerance of cultivated rice to keep its yield stable and high, and to widen its planting range of space and time, on the other hand, it is helpful to elucidate the physiological & metabolic basis and the molecular mechanism underlying its strong cold tolerance using ratoon ‘Chaling’ CWR as material to deepen the understanding of cold tolerance of *Oryza* plants.

As showed in this study that the ratoon ‘Chaling’ CWR is extremely cold-tolerant, and it can safely overwinter in Changsha, however the cold tolerance of the seedlings germinating from ‘Chaling’ CWR seeds is not extremely strong [13], for they cannot safely overwinter in Changsha. This phenomenon has also been observed in the cold tolerance studies of other wild rice accessions [19]. Based on these studies, it is inferred that there is no linkage relationship regarding cold tolerance among different developmental stages. What is the cause of this difference about cold tolerance is currently unclear and may be complicated.

## Conclusion

Ratoon ‘Chaling’ CWR possesses strong cold tolerance and certain freezing tolerance, and it can safely overwinter under the natural conditions in Changsha (28° 22′ N), Hunan province, China, which is a further and colder northern place than its habitat. Even if ratoon ‘Chaling’ CWR suffers a long-term cold stress (the average daily mean temperature of 22 consecutive days is − 1.0 °C, the average daily minimum temperature is − 1.7 °C, the average daily maximum temperature is 2.3 °C, and the extreme low temperature is − 4.7 °C in Changsha), it can still survive naturally.

## Methods

### Plant materials

CWR materials include ‘Chaling’ CWR (26° 50′ N), ‘Dongxiang’ CWR (28° 14′ N), ‘Jiangyong’ CWR (25° 41′ N), ‘Liujiang’ CWR (24° 18′ N), ‘Fusui’ CWR (22°25′ N), ‘Hepu’ CWR (21° 38′ N), and ‘Hainan’ CWR (18° 73′ N), and CCR materials tested are Nipponbare (*japonica*), Qiuguang (*japonica*), Dongnong 416 (*japonica*), 01 Zhao 001 (*javanica*), 01 Zhao 085 (*javanica*), 01 Zhao 092 (*javanica*), Nanjing 11 (*indica*), Guangluai 4 (*indica*), 93–11 (*indica*), Liangyou Peijiu (*indica*), and Peiai 64 S (*indica*). Among them, all the materials except *javanica* rice materials are provided by the College of Life Sciences, Hunan Normal University. *Javanica* rice materials is provided by Dr. Xiao Guoying, engaged in the Institute of Subtropical Agriculture, Chinese Academy of Sciences (Dr. Xiao obtained the *javanica* rice materials from the International Rice Research Institute).

### Field test method for cold tolerance evaluation of ratoon ‘Chaling’ CWR

The field trial for cold tolerance evaluation of ratoon ‘Chaling’ CWR was conducted in a paddy field in the Institute of Subtropical Agriculture, Chinese Academy of Sciences from June 2007 to May 2008. Each material is planted in 3 plots randomly arranged in the field, and 20 plants are planted in each plot with a plant spacing of 33 cm × 33 cm, thus 60 plants in total for every material. The CWR materials such as ‘Chaling’ CWR are transplanted with ratoon seedlings, while CCR materials are transplanted with seed seedlings at the age of 25 days. The transplanting date is June 25, 2007. Conventional water and fertilizer management and pest control were performed. When all the CWR and CCR materials headed and their panicles got seeds (only Hainan wild rice can not head at Changsha), their shoots were cut but keeping the culm height 30 cm for regeneration, then let them overwinter in the natural conditions of Changsha. The survival rates of ratoon CWR and CCR were determined after the air temperature rises in April and May next year.

### Pot experiment method for cold tolerance evaluation of ratoon ‘Chaling’ CWR

The pot experiment for cold tolerance evaluation of ratoon ‘Chaling’ CWR was conducted at the College of Life Sciences, Hunan Normal University from June 2009 to May 2010 and from June 2010 to May 2011, respectively. Five pots of each material was planted (the pot is filled with wet paddy soil whose dry weight is 3.5 kg), and 2 plants were planted per pot, therefore, a total of 10 plants were planted for every material. The transplanting dates were June 25, 2009 and June 25, 2010, respectively. The transplanting method, water and fertilizer management, pest control, and the measured indicator were the same as in the field test.

### Statistical analysis

The number of plants before winter and after winter in Table 2 are means of plants grown in 3 independent plots. One way ANOVA and Duncan’s multiple range tests were used to determine the significant differences among means obtained from ratoon ‘Chaling’ CWR and the other ratoon CWR or ratoon CCR at the 5% level. All calculations were performed using the statistical software of IBM SPSS Statistics 19.

## Data Availability

All data generated or analysed during this study are included in this published article.

## Abbreviations

CWR: Common wild rice
CCR: Common cultivated rice
QTL: Quantitative trait locus/loci
SD: Standard deviation
